# Influence of Aluminium and EGCG on Fibrillation and Aggregation of Human Islet Amyloid Polypeptide

**DOI:** 10.1155/2016/1867059

**Published:** 2016-12-15

**Authors:** Zhi-Xue Xu, Qiang Zhang, Gong-Li Ma, Cong-Heng Chen, Yan-Ming He, Li-Hui Xu, Yuan Zhang, Guang-Rong Zhou, Zhen-Hua Li, Hong-Jie Yang, Ping Zhou

**Affiliations:** ^1^State Key Laboratory of Molecular Engineering of Polymers, Department of Macromolecular Science, Fudan University, Shanghai 200433, China; ^2^Yueyang Hospital of Integrated Traditional Chinese and Western Medicine, Shanghai University of Traditional Chinese Medicine, Shanghai 200437, China; ^3^Collaborative Innovation Center of Chemistry for Energy Material, Shanghai Key Laboratory of Molecular Catalysis & Innovative Materials, Department of Chemistry, Fudan University, Shanghai 200433, China; ^4^Department of Medicine, St Vincent's Hospital, The University of Melbourne, Fitzroy, VIC 3065, Australia

## Abstract

The abnormal fibrillation of human islet amyloid polypeptide (hIAPP) has been implicated in the development of type II diabetes. Aluminum is known to trigger the structural transformation of many amyloid proteins and induce the formation of toxic aggregate species. The (−)-epigallocatechin gallate (EGCG) is considered capable of binding both metal ions and amyloid proteins with inhibitory effect on the fibrillation of amyloid proteins. However, the effect of Al(III)/EGCG complex on hIAPP fibrillation is unclear. In the present work, we sought to view insight into the structures and properties of Al(III) and EGCG complex by using spectroscopic experiments and quantum chemical calculations and also investigated the influence of Al(III) and EGCG on hIAPP fibrillation and aggregation as well as their combined interference on this process. Our studies demonstrated that Al(III) could promote fibrillation and aggregation of hIAPP, while EGCG could inhibit the fibrillation of hIAPP and lead to the formation of hIAPP amorphous aggregates instead of the ordered fibrils. Furthermore, we proved that the Al(III)/EGCG complex in molar ratio of 1 : 1 as Al(EGCG)(H_2_O)_2_ could inhibit the hIAPP fibrillation more effectively than EGCG alone. The results provide the invaluable reference for the new drug development to treat type II diabetes.

## 1. Introduction

**Figure 1:**
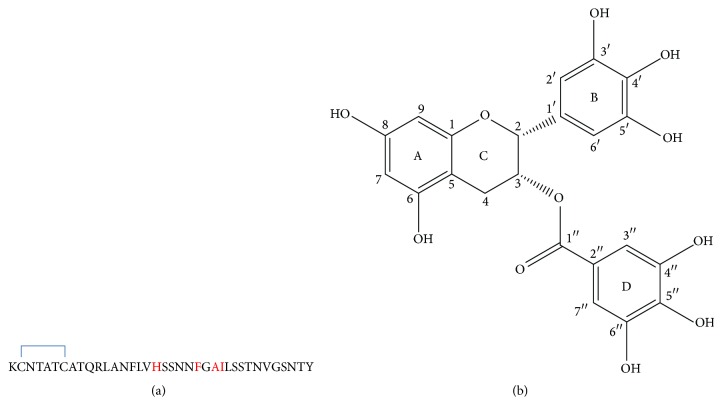
(a) Sequence of hIAPP. (b) Structure of EGCG.

A variety of degenerative diseases including Alzheimer's disease, Parkinson's disease, and type II diabetes are pathologically characterized by amyloid deposits [1–4]. Human islet amyloid polypeptide (hIAPP), a 37-amino acid residue polypeptide (Figure 1(a)), has the propensity to form the oligomers and fibrils [5, 6] which are thought to be toxic to the pancreatic islet *β*-cells [7] and play the pathological role in the development of type II diabetes [8]. Therefore, inhibiting the formation of toxic hIAPP oligomers and fibrils could be a potential therapeutic strategy for the treatment of type II diabetes.

Earlier studies showed that metal ions such as aluminum, zinc, copper, iron, manganese, and calcium are implicated in the fibrillation and aggregation of amyloidogenic proteins [9–15]. These metal ions can cause the amyloid peptide rapid precipitation and alter the morphology of the final aggregates [10]. The aluminum concentration in serum was found to be 22.8 ± 18.1 *μ*g/L in diabetic patients, significantly higher than 11.4 ± 5.1 *μ*g/L in healthy subjects [16]. What is more, aluminum was demonstrated to affect the formation rate and morphology of proislet amyloid polypeptide (the precursor peptide of hIAPP) fibers [17]. However, how these metal ions affect the fibrillation and aggregation of hIAPP has not been elucidated clearly. Therefore, investigation of these metal ions promoting the protein folding and their potential inhibitors would be important for better understanding the hIAPP fibrillation in pancreatic islet *β*-cells and developing the novel therapeutic drugs for type-II diabetes. Thioflavin T (ThT) fluorescence is one of the most widely used experimental approaches to probe the kinetics of *β*-sheet enriched fibrillation with high sensitivity [18] while NMR spectroscopy can monitor the aggregation of protein on the fact that the solidification of protein makes the resonance peaks broadened and even covered up in the noise background for the specific amino acid residues on the atomic level [19].

To date, some of hIAPP fibrillation inhibitors such as aromatic compounds, short peptides, and ligand-metal-chelate complexes have been studied* in vitro* and* in vivo* [3, 20–26]. Among those potential inhibitors, (−)-epigallocatechin gallate (EGCG, Figure 1(b)), an aromatic compound with many hydroxyl groups and abundantly present in green teas, can inhibit the fibrillation of amyloid *β* peptide, *α*-synuclein, semen-derived enhancer of virus infection, and hIAPP [27–31] and even remodel the formed amyloid fibrils [32, 33]. Previous studies have shown that EGCG can bind to hIAPP monomers, resulting in the formation of amorphous aggregates instead of ordered amyloid fibrils [34, 35]. In addition, EGCG has also inhibitory potency on the metal-induced amyloid deposits [36, 37].

In this work, we sought to study the chelation of Al(III) and EGCG by using various spectroscopic experiments and quantum chemical calculations and also investigate the influence of both Al(III) and EGCG individually on hIAPP fibrillation and aggregation as well as their complexation on this process by using spectroscopic and microscopic techniques. The results may provide the meaningful references for the new drug development of the diabetes treatment.

## 2. Materials and Methods

### 2.1. Materials

Human islet amyloid polypeptide (hIAPP) was synthesized by Chinese Peptide, China. EGCG was purchased from TCI, Japan. Aluminum chloride (AlCl_3_) was purchased from Sinopharm, China. Hexafluoroisopropyl alcohol (HFIP) was purchased from Aladdin, China. Deuterated water (D_2_O) was purchased from J&K, China. The purity of hIAPP was greater than 95% and the molecular weight was confirmed by ESI-MS and MALDI-TOF-MS.

### 2.2. Preparation of Samples

#### 2.2.1. Preparation of Peptide Stock Solutions

hIAPP stock solutions were prepared by dissolving the hIAPP in HFIP. The solutions were sonicated at room temperature for 3 min and then filtered through a 0.45 *μ*m filter to remove insoluble materials. The stock solutions (800 *μ*M) were stored at 4°C.

#### 2.2.2. Preparation of Al(III)/EGCG Samples

50 *μ*M EGCG aqueous solution was mixed with 0, 1.7, 4.2, 6.7, 8.3, 12.5, 16.7, 25, 33.3, 41.7, 50, 58.3, 66.7, 75, 100, 125, 133.3, and 150 *μ*M AlCl_3_ for UV-vis spectroscopic study. 150 *μ*M EGCG aqueous solution was mixed with 0, 5, 12.5, 20, 25, 37.5, 50, 75, 100, 125, 150, 175, 200, 225, 300, 375, 400, and 450 *μ*M AlCl_3_ for fluorescence spectroscopic study. 10 mM EGCG aqueous solution was mixed with or without 10 mM AlCl_3_ for electrospray ionization mass spectroscopic study. 10 mM EGCG was mixed with 0, 0.5, 1, 2, 3, 4, 6, 7, 8, 10, 12, 14, 16, 18, 20, 25, and 30 mM AlCl_3_ in D_2_O for ^1^H NMR spectroscopy.

#### 2.2.3. Preparation of Al(III)/hIAPP Samples

hIAPP stock solutions were added to D_2_O solution with or without 100 *μ*M AlCl_3_ to give an hIAPP concentration of 100 *μ*M for ^1^H NMR spectroscopy.

#### 2.2.4. Preparation of EGCG/hIAPP Samples

hIAPP stock solutions were added to D_2_O with 0, 1, and 5 mM EGCG to give final concentration of hIAPP at 100 *μ*M for study the influence of EGCG on hIAPP using ^1^H NMR spectroscopy. In addition, hIAPP stock solutions were added to D_2_O with 1 mM EGCG to give final concentration of hIAPP at 0, 20, and 100 *μ*M for the study of hIAPP influence on EGCG using ^1^H NMR spectroscopy.

#### 2.2.5. Preparation of Samples for Kinetic Studies

For the fibrillation kinetic studies, hIAPP stock solutions were mixed with equivalent ThT aqueous solutions, various volumes of 1 mM AlCl_3_ aqueous solutions, and various volumes of 15 mM EGCG fresh aqueous solutions and then diluted with PBS aqueous solution (20 mM and pH = 7.4). The final concentrations of hIAPP and ThT were both 16 *μ*M for ThT fluorescence study. Three replicates of each sample were prepared in a 96-well plate (NUNC number 237108) with volume of 200 *μ*L per well.

For the aggregation kinetic studies, hIAPP stock solutions were added to 6 mM PBS D_2_O solution with or without 12.5 *μ*M AlCl_3_ or 12.5 *μ*M EGCG to give an hIAPP concentration of 100 *μ*M for ^1^H NMR study.

For nucleation kinetic studies, 16 *μ*M hIAPP was incubated in 6 mM PBS with or without 2 *μ*M EGCG, 2 *μ*M AlCl_3_, or mixture of 2 *μ*M EGCG and 2 *μ*M Al(III). The samples were filtered through a 0.45 *μ*m filter to remove dust for dynamic light scattering study.

#### 2.2.6. Preparation of Samples for TEM Observation

16 *μ*M hIAPP was incubated in 20 mM PBS with or without 2 *μ*M EGCG, 2 *μ*M AlCl_3_, or mixture of 2 *μ*M EGCG and 2 *μ*M Al(III) at 37°C for 2 h. 10 *μ*L incubated solutions were placed on 300 mesh formvar-coated copper grids for 10 min and dried at room temperature for 2 h. The samples were gently rinsed with water to remove the PBS salt in the samples and then stained with 2% fresh uranyl acetate for another 2 min. The grids were blotted on filter paper and dried in vacuum overnight.

### 2.3. Methods

#### 2.3.1. UV-Vis Spectroscopy

UV-vis spectra were recorded by Lambda 35 UV-vis spectrometer (Perkin-Elmer, USA) at 25°C with a 1.0 cm path length quartz cell at wavelength from 250 to 375 nm with resolution of 1 nm. All UV-vis experiments were repeated three times and averaged with standard deviation (mean ± SD).

#### 2.3.2. Fluorescence Spectroscopy

The chelation of EGCG with Al(III) was studied by fluorescence spectroscopy using QM 40 fluorescence spectrometer (PTI, USA) at 25°C with a 1.0 cm path length quartz cell at the excitation wavelength of 324 nm and emission wavelength from 250 to 375 nm with resolution of 1 nm. Experiments were repeated three times and averaged with standard deviation (mean ± SD).

The fibrillation of hIAPP was monitored by ThT fluorescence using a fluorescence plate reader (ThermoFisher, USA) at 37°C at the excitation wavelength of 450 nm and emission wavelength of 480 nm with 1-minute interval, and the fluorescence intensity of samples was referred to that of corresponding ThT free solutions. Experiments were replicated three times and averaged.

#### 2.3.3. Electrospray Ionization Mass Spectroscopy

Mass spectrum was recorded by electrospray ionization mass spectrometry (ESI-MS) using a LTQ ion trap spectrometer (ThermoFisher, USA) in positive ion mode with capillary temperature of 250°C and spray voltage of 2 kV. Samples were injected into the ion source, using nitrogen as the drying gas.

#### 2.3.4. ^1^H NMR Spectroscopy

^1^H NMR spectra were recorded using a Bruker Avance III HD 400 MHz spectrometer (Bruker BioSpin International, Germany) at 25°C with 48 transients and 3.0 s pulse delay between each transient. For Al(III)/hIAPP interaction study, ^1^H NMR spectra were recorded after samples were incubated for 0 min, 40 min, and 17 h. For the aggregation study of hIAPP, the kinetic measurements were monitored with a 6-minute interval.

#### 2.3.5. Transmission Electron Microscopy

The images of hIAPP aggregates were recorded by Tecnai G2 20 TWIN transmission electron microscope (FEI, USA) at 200 kV under vacuum. The point resolution was 0.27 nm, and the tracer resolution was 0.14 nm.

Image J software was used to measure the diameters of amyloid fibrils and amorphous aggregates in the TEM images. Mean diameters of fibrils and amorphous aggregates were calculated from 40 randomly selected fibrils or aggregates in each group. Results are expressed as mean ± SD [38].

#### 2.3.6. Dynamic Light Scattering

Nucleation of hIAPP was monitored by dynamic light scattering (DLS) using a Zeta-Sizer (Malvern Instruments, UK) at 37°C with a 1.0 cm path length quartz cell. *Z*-average sizes of particles were monitored at *λ* = 633 nm with a 30-second interval (slit width = 1 nm). Data with PDI > 0.4 were abandoned for the reliability.

#### 2.3.7. Logistic Equation

ThT fluorescence and ^1^H NMR integrated intensity of hIAPP on the incubation time were fitted on logistic equation as follows [10, 35]:

(1) $$\begin{array}{c} I=\frac{I_{max}}{1+e^{\pm \left(t-t_{1/2}\right)k}}, \end{array}$$

where *I*_max_ is the maximum fluorescence values or the maximum integration values of ^1^H NMR resonances; *t*_1/2_ is the time required to reach half *I*_max_; and *k* is an apparent first-order rate constant for addition of hIAPP to form the fibers. The symbol “−” of “±” was selected for the ThT fluorescence assay with the increased signal intensity and “+” for the ^1^H NMR assay with the decreased signal intensity. As reported [10], lag-time *t*_0_, the time predicted by nucleation dependent polymerization theory before detectable amyloid aggregation occurring, was described by *t*_0_ = *t*_1/2_ − 2/*k*.

#### 2.3.8. Kolmogorov-Johnson-Mehl-Avrami Equation

*Z*-average size of hIAPP on the incubation time was fitted on Kolmogorov-Johnson-Mehl-Avrami (KJMA) equation as follows [39]:

(2) $$\begin{array}{c} D^{3}=D_{0}^{3}+\left(\;D_{lim}^{3}-D_{0}^{3}\;\right)\left(\;1-e^{-kt^{n}}\;\right), \end{array}$$

where *D* is the *Z*-average diameter of aggregates, *D*_0_ is the initial *Z*-average diameter of aggregates, *D*_lim_ is the final *Z*-average diameter of aggregates, *k* is related to the growth rate of aggregates, and *n* is called Avrami exponent.

#### 2.3.9. Quantum Chemical Calculations of Al(III)/EGCG Complex

Quantum chemical calculations were all performed at the density functional theory level using the hybrid meta-GGA M06-2x functional [40], which has been proven to give reliable results for the structural and energetic properties and binding free energy of noncovalent systems [41]. Based on ^1^H NMR results, A-ring was the major binding site of EGCG to Al(III). Therefore, to reduce the computational costs, a “fragmental EGCG” without B-ring and D-ring was used as model with 6-31+G(d,p) basis set. Full geometry optimization was carried out in the water solution which was modelled by the polarizable continuum solvation model (IEFPCM) [42] with radii and nonelectrostatic terms for Truhlar and coworkers' SMD solvation model [43]. This solvation model is one of the most reliable models in predicting solvation free energies. The dielectric constant used for water is 78.3553. The convergence criteria used for the geometry optimization are 4.50 × 10^−4^ a.u. for gradients and 1.80 × 10^−3^ a.u. for displacements. Harmonic vibrational analyses were carried out to verify if the optimized structure is a local minima or a first-order transition state and to provide zero-point vibrational energy corrections and thermal corrections to various thermodynamic properties. All the calculations were performed by using the Gaussian 09 program.

## 3. Results and Discussion

### 3.1. Chelation Study of EGCG with Al(III)

**Figure 2:**
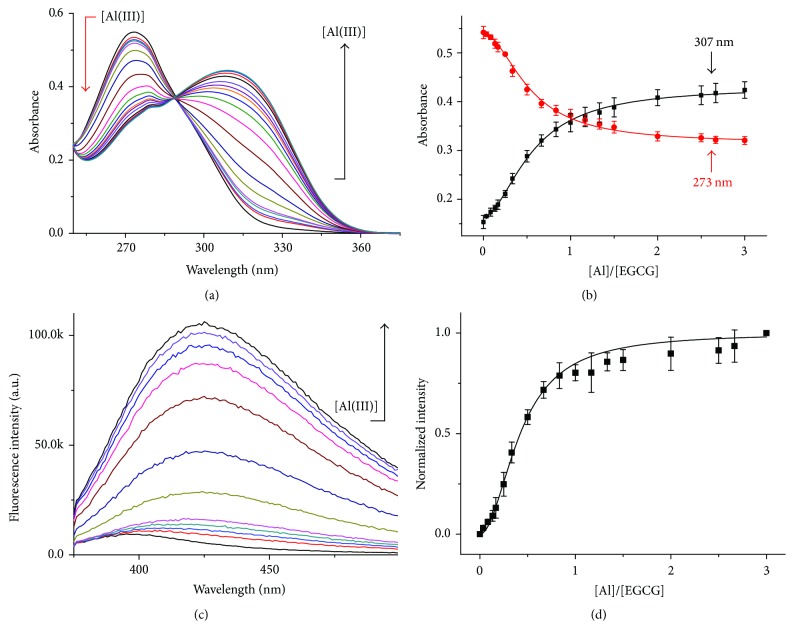
Chelation of Al(III) and EGCG. (a) UV spectra of 50 *μ*M EGCG in the presence of Al(III) at various concentrations from 0 to 150 *μ*M. (b) Dependence of absorbance of 50 *μ*M EGCG at 273 nm (red) and 307 nm (black) on the molar ratios of Al(III) to EGCG from 0 to 3. The values represent mean ± SD (*n* = 3). (c) Fluorescence spectra of 150 *μ*M EGCG as Al(III) concentration increased from 0 to 450 *μ*M. (d) 150 *μ*M EGCG fluorescence intensity at 425 nm on molar ratio of Al(III) to EGCG. The intensity values were normalized to 150 *μ*M EGCG with 450 *μ*M Al(III). The values represent mean ± SD (*n* = 3).

Before study of influence of Al(III) and EGCG on hIAPP, we firstly investigated the structure of Al(III)/EGCG complex by using spectroscopic methods including UV-visible absorption spectroscopy, fluorescence emission spectroscopy, electrospray ionization mass spectroscopy, and ^1^H NMR. UV-vis spectra of 50 *μ*M EGCG with titration of Al(III) in aqueous solution were recorded and an absorption peak was found to be red-shifted from 273 to 307 nm as the molar ratio of Al(III) to EGCG increased from 0 to 3 (Figures 2(a) and 2(b)), which indicated that Al(III) interacted with EGCG. Furthermore, the fluorescence spectra of 150 *μ*M EGCG with titration of Al(III) in aqueous solution were recorded. It was found that a new broad peak appeared at 425 nm and the intensity of this peak was increased with the increased molar ratio of Al(III) to EGCG from 0 to 3 (Figures 2(c) and 2(d)), which indicated that Al(III) chelated EGCG and formed a new complex.

Both UV spectra (Figure 2(b)) and fluorescence spectra (Figure 2(d)) of EGCG titrated with Al(III) demonstrated that Al(III)/EGCG complex was in molar ratio of 1 : 1. To clarify the structure of Al(III)/EGCG complex, EGCG and Al(III)/EGCG mixture in molar ratio of 1 : 1 was analyzed by ESI–MS in Figure S1 in Supplementary Material available online at http://dx.doi.org/10.1155/2016/1867059. The assignments of ESI–MS spectra were shown in Table S1. The results also demonstrated that the major molar ratio of Al(III)/EGCG complex was 1 : 1 at *m*/*z* = 518(Al^3+^(EGCG − 2H^+^) + 2H_2_O), which was identical to the results from UV-vis and fluorescence spectroscopy.

**Figure 3:**
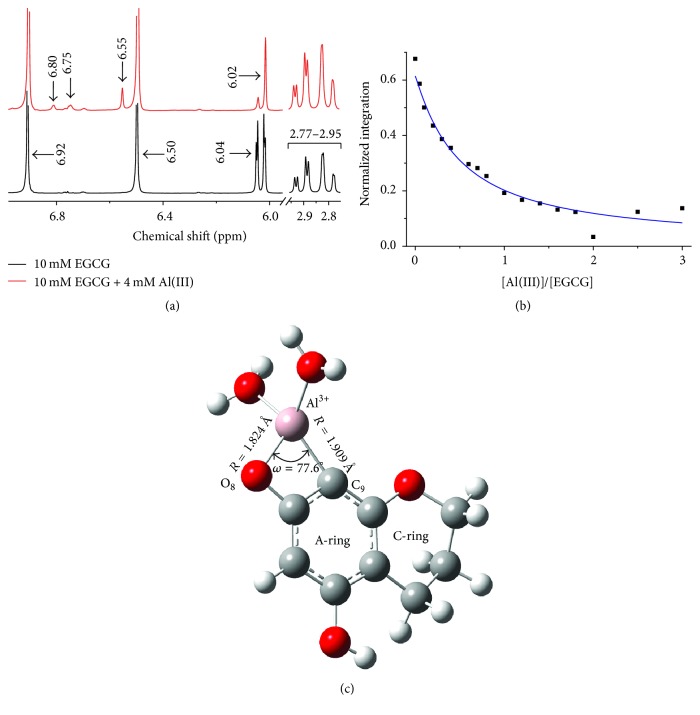
(a) ^1^H NMR spectra of 10 mM EGCG without (bottom) and with (top) 4 mM Al(III). (b) The ^1^H resonance integration at *δ* = 6.04 ppm of C-9 in 10 mM EGCG on the [Al(III) ]/[EGCG] ratio as the titration of Al(III) from 0 to 30 mM. The values were normalized to ^1^H integration within 2.77 to 2.95 ppm of C-4. (c) Illustration of Al(EGCG)(H_2_O)_2_ complex 1 (AE-1). Red, gray, pink, and white balls represent oxygen, carbon, aluminum, and hydrogen atoms, respectively. C_9_ and O_8_ in A-ring of EGCG and two more O atoms from H_2_O coordinate to the aluminum.

To characterize the coordination sites of Al(III) to EGCG, ^1^H NMR of EGCG with titration of Al(III) in D_2_O was measured in Figure 3(a) and Figure S2. The assignments of NMR spectra were shown in Table S2 [44, 45]. As shown in Figure 3(a), weak aromatic proton signals were observed at *δ* = 6.55 ppm close to the B-ring proton signal *δ* = 6.50 ppm and at *δ* = 6.75 as well as 6.80 ppm close to the D-ring proton signal *δ* = 6.92 ppm of EGCG after the addition of Al(III), which suggested the interaction occurring between gallate group of EGCG and Al(III). The signal at *δ* = 6.04 ppm assigned to C_9_ of EGCG decreased significantly as molar ratio of Al(III) to EGCG increased before 1 : 1, while decreasing slowly afterward (Figure 3(b)), which indicated that C_9_ of EGCG was the main binding site with Al(III), and the Al(III)/EGCG complex was dominantly in ratio of 1 : 1. Combining the ESI-MS results, we suggested that one aluminum cation most probably chelated one EGCG molecule at C_9_ and O sites along with two more O atoms from two water molecules to form a new complex of Al(EGCG)(H_2_O)_2_.

To further clarify the structure of Al(III)/EGCG complex, the quantum chemical calculation was used. Based on ^1^H NMR and ESI-MS results, we speculated that there are two possible structures of Al(III)/EGCG complex. One structure was named as AE-1 with Al(III) coordinating to C_9_ atom and O_8_ in A-ring from one EGCG molecule as well as two more oxygen atoms from two water molecules as shown in Figure 3(c) and Figure S3. The other structure was named AE-2 with Al(III) coordinating to C_9_ atom in A-ring and -O- atom in C-ring from one EGCG as well as two more oxygen atoms from two water molecules as shown in Figure S4 and Figure S5. The binding free energies of these complexes were calculated according to the following equation:

(3) ΔGbinding=Gcomplex+GH+−GAl3+−GEGCG·H−1−2GH2O.

It was demonstrated in Table S3 that the binding free energy of AE-1 and AE-2 at 0 K is −0.43 and −0.39 a.u., respectively, indicating that the structure of AE-1 is more favourable than that of AE-2. The Al(III)/EGCG complex of Al(EGCG)(H_2_O)_2_ in AE-1 structure was formed in bond distances of 1.824 Å and 1.909 Å for Al-O_8_ and Al-C_9_, respectively, and bond angle of 77.6° for O_8_-Al-C_9_. The Al(III)/EGCG complex of Al(EGCG)(H_2_O)_2 _in AE-2 structure was formed in bond distances of 1.895 Å and 1.926 Å for Al-O in C-ring and Al-C_9_ in A-ring of EGCG, respectively, and bond angle of 74.7° for O-Al-C_9_.

### 3.2. Self-Fibrillation and Self-Aggregation of hIAPP

**Figure 4:**
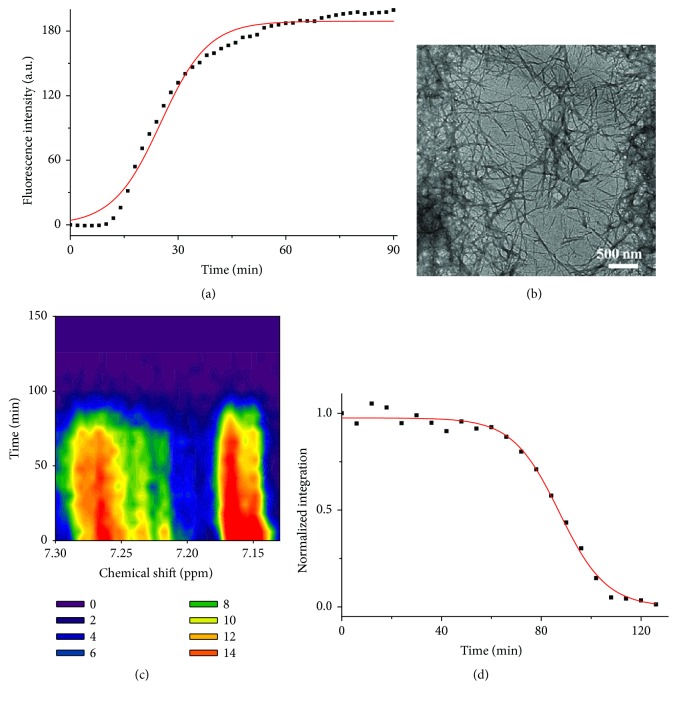
Self-aggregation of hIAPP. (a) Fibrillation kinetics of 16 *μ*M hIAPP in 20 mM PBS at pH 7.4 under 37°C monitored by ThT fluorescence. The values represent mean (*n* = 3). Solid lines represent the curves fitted using (1). (b) TEM image of 16 *μ*M hIAPP incubated in 20 mM PBS for 2 h. (c) Contour plots of ^1^H NMR spectra within chemical shift from 7.13 to 7.3 ppm for 100 *μ*M hIAPP incubated in 6 mM PBS at pH 7.4 and 25°C. (d) Aggregation kinetics of integration resonance intensity of ^1^H NMR spectra from 7.13 to 7.3 ppm for 100 *μ*M hIAPP. Solid lines represent the curves fitted using (1). The integration values were normalized to the initial hIAPP alone data.

To evaluate the effect of EGCG and Al(III) on hIAPP fibrillation and aggregation, we investigated these processes by ThT fluorescence spectroscopy, microscopy, and ^1^H NMR. 16 *μ*M hIAPP was incubated in 20 mM PBS at pH 7.4 under 37°C for 3 h.  Figure 4(a) shows the fibrillation of hIAPP in a typical sigmoidal time-dependent manner, monitored by ThT fluorescence spectrum, which indicated the self-fibrillation of hIAPP following a nucleation mechanism [46].

The aggregates morphologies of 16 *μ*M hIAPP incubated for 2 h were observed by TEM (Figure 4(b)). Some single fibrils in average diameter of 21 ± 5 nm and some fibril bundles in average diameter of 73 ± 16 nm were observed in hIAPP sample. The results demonstrated that hIAPP dominantly self-assembled into fibrils.

Furthermore, the peptide aggregation was also investigated by real-time NMR as shown in Figure 4(c). 100 *μ*M hIAPP was incubated in 6 mM PBS at pH 7.4 under 25°C. It is found in Figure 4(d) that the normalized ^1^H NMR integration intensity of aromatic residues in hIAPP within chemical shift from 7.13 to 7.30 ppm was decreased in a typical sigmoidal manner and even no signals were observed after 3 h incubation because the large aggregates were formed. This phenomenon was also observed by other authors for the studies of amyloid fibrillation [47, 48] and amorphous aggregation [49]. The reason is the solidification of protein making the resonance peaks broadened and even covered up in the noise background. Our results are in good agreement with those reported in the literature [50].

### 3.3. Influence of Al(III) on the Fibrillation and Aggregation of hIAPP

**Figure 5:**
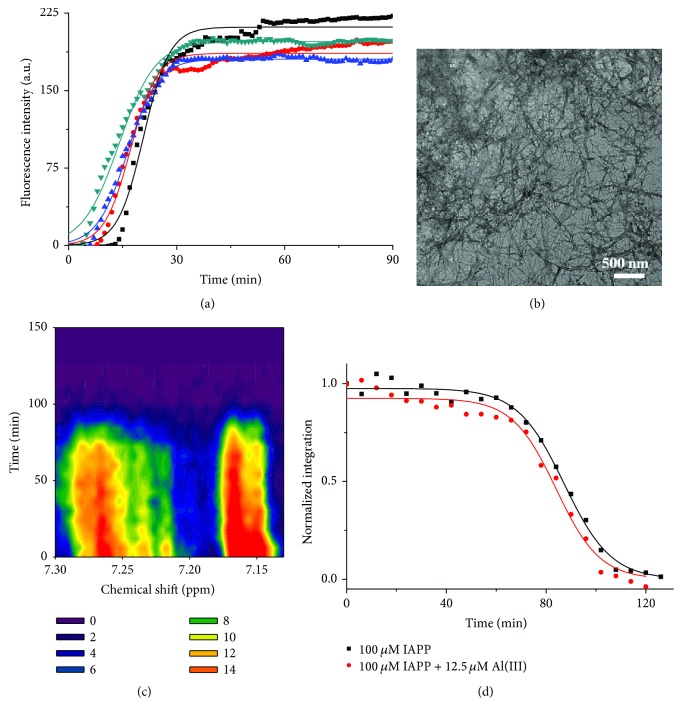
The influence of Al(III) on the fibrillation and aggregation of hIAPP. (a) Kinetics of 16 *μ*M hIAPP fibrillation with 0 (■), 2 (●), 8 (▲), and 64 (▼) *μ*M Al(III) in 20 mM PBS at pH 7.4 under 37°C, monitored by ThT fluorescence. The values represent mean (*n* = 3). Solid lines represent the curves fitted using (1). (b) TEM image of 16 *μ*M hIAPP incubated in 20 mM PBS with 2 *μ*M Al(III) for 2 h. (c) Contour plots of ^1^H NMR spectra within chemical shift from 7.13 to 7.3 ppm for 100 *μ*M hIAPP incubated in 6 mM PBS at pH 7.4 and 25°C on incubation time with 12.5 *μ*M Al(III). (d) Aggregation kinetics of integrated resonance intensity of ^1^H NMR spectra from 7.13 to 7.3 ppm of 100 *μ*M hIAPP without (■) and with (●) 12.5 *μ*M Al(III). Solid lines represent the curves fitted using (1). The integration values were normalized to the initial hIAPP alone data.

Figure 5(a) shows the fibrillation of hIAPP with titration of Al(III) in time-dependent manner, monitored by ThT fluorescence spectrum. It is found in Table S4 and Figure S6 that the lag-time *t*_0_ decreased from 13.6 ± 0.5 min to 4.0 ± 0.4 min with the increased concentration of Al(III) from 0 to 64 *μ*M on (1), which indicated that Al(III) promoted the fibril nucleation of hIAPP.

The aggregates morphologies of 16 *μ*M hIAPP incubated with 2 *μ*M Al(III) for 2 h were observed by TEM (Figure 5(b)). It is found in Figure 5(b) that the predominant fibrils in Al(III) treated sample were twisted in average diameter of about 33 ± 13 nm. The fiber distribution of Al(III) treated sample was much more intensive than that of untreated sample (Figure 4(b)). The results demonstrated that Al(III) stimulated the hIAPP fibril nucleation.

Furthermore, the peptide aggregation interfered by Al(III) was also investigated by real-time ^1^H NMR as shown in Figure 5(c). 100 *μ*M hIAPP was incubated with 12.5 *μ*M Al(III) in 6 mM PBS at pH 7.4 under 25°C. It was also found in Figure 5(d) and Table S5 that the lag-time *t*_0_ was decreased from 69 ± 1 to 66 ± 2 min on (1) with the addition of Al(III), which indicated that Al(III) slightly accelerated the process of hIAPP fibril nucleation and aggregation.

It was reported [15, 26] that Al(III)-induced fibrillation and aggregation involve the octahedral Al-O and Al-N bonding between aluminum and amyloidogenic proteins, forming the O(N)-Al(III)-O(N) cross-links between protein monomers and leading to the amyloid nucleation and aggregation.

**Figure 6:**
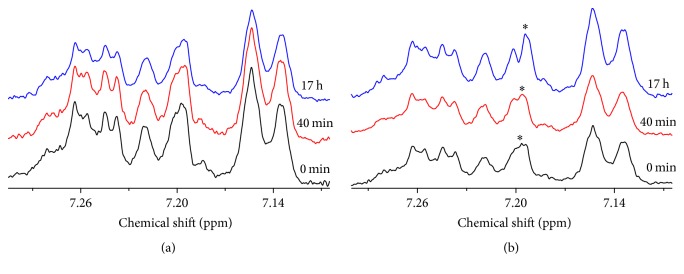
The interaction between hIAPP and Al(III). (a) and (b) are ^1^H NMR spectra of 100 *μ*M hIAPP without and with 100 *μ*M Al(III), respectively, after 0 min (black), 40 min (red), and 17 h (blue) incubation. The asterisks show the *H*_*ε*_ resonance of His18.

In order to explore the interaction between hIAPP and Al(III) in detail, ^1^H NMR spectra of 100 *μ*M hIAPP incubated with or without high concentration of Al(III) at 100 *μ*M were measured for 0 min, 40 min, and 17 h as shown in Figure 6. Only signals corresponding to the aromatic residues of hIAPP were changed and those of other residues were nearly unchanged. The assignments of ^1^H NMR spectra of aromatic resonances of hIAPP were listed in Table S6. We found that the resonance at *H*_*δ*_ in the imidazole ring of histidine 18 (His18) of hIAPP [51] without Al(III) present was nearly unchanged (Figure 6(a)), while addition of Al(III) made the peak gradually split into doublet as incubation time (Figure 6(b)). Previous studies have shown that His18 plays a key role in the fibrillation of hIAPP, especially in the Zn(II) coincubated fibrillation process [52–54]. Zn(II) preferentially binds six hIAPP monomers to form a hIAPP hexamer cluster and thus inhibits the formation of mature fiber, for its strong binding affinity to hIAPP monomers. Al(III), its binding affinity much lower than that of Zn(II), may bind stepwise to the nitrogen in imidazole ring of His18 in hIAPP, forming the coordination bond of Al-N and further the cross-link of His18-Al(III)-His18 between two hIAPP monomers [15, 26], which might promote the fibril nucleation of hIAPP.

### 3.4. Influence of EGCG on the Fibrillation and Aggregation of hIAPP

**Figure 7:**
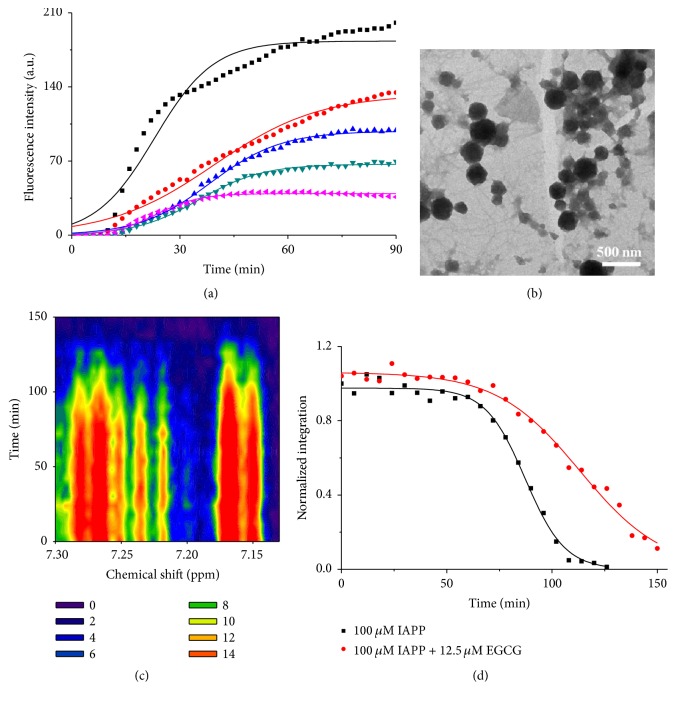
The influence of EGCG on the fibrillation and aggregation of hIAPP. (a) Kinetics of 16 *μ*M hIAPP fibrillation with 0 (■), 2 (●), 8 (▲), 16 (▼), and 32 (◂) *μ*M EGCG in 20 mM PBS at pH 7.4 under 37°C monitored by ThT fluorescence. The values represent mean (*n* = 3). Solid lines represent the curves fitted using (1). (b) TEM image of 16 *μ*M hIAPP incubated in 20 mM PBS with 2 *μ*M EGCG for 2 h. (c) Contour plots of ^1^H NMR spectra within chemical shift from 7.13 to 7.3 ppm for 100 *μ*M hIAPP incubated in 6 mM PBS at pH 7.4 and 25°C on incubation time with 12.5 *μ*M EGCG. (b) Aggregation kinetics of integrated resonance intensity of ^1^H NMR spectra from 7.13 to 7.3 ppm of 100 *μ*M hIAPP without (■) and with (●) 12.5 *μ*M EGCG. Solid lines represent the curves fitted using (1). The integration values were normalized to the initial hIAPP alone data.

To examine the inhibition effect of EGCG on the hIAPP fibrillation, 16 *μ*M hIAPP was incubated with various concentrations of EGCG from 0 to 32 *μ*M. The fibrillation kinetics of hIAPP were also monitored by ThT fluorescence. It is found in Figure 7(a) that both fibrillation processes of hIAPP with or without EGCG were in time-dependent sigmoidal manner, but the maximum fluorescence intensity *I*_max_ decreased from 186.9 ± 3.1 to 42.2 ± 0.4 on (1) with the increased EGCG concentration (Table S7 and Figure S7), which indicated that amount of the amyloid fibers was reduced significantly as the interference of EGCG.

The aggregates morphologies of 16 *μ*M hIAPP incubated with or without 2 *μ*M EGCG for 2 h were observed by TEM in Figure 7(b). It is found that most of hIAPP aggregates with EGCG present were spherical in average diameter of 184 ± 68 nm with a few fibrils in average diameter of about 29 ± 9 nm, while those without EGCG were typical fibrils as shown in Figure 4(b), which suggested that EGCG promoted the hIAPP forming the amorphous aggregates instead of typical fibrils.

Furthermore, the influence of EGCG on hIAPP aggregation was also investigated by real-time ^1^H NMR. The ^1^H NMR results of 100 *μ*M hIAPP incubated with 12.5 *μ*M EGCG in 6 mM PBS at pH 7.4 under 25°C were shown in Figure 7(c) within chemical shift from 7.13 to 7.30 ppm where the aromatic resonances of hIAPP were present, but not resonances of EGCG. It is found that the integration resonance intensity of aromatic region decreased in time-dependent sigmoidal manner with or without EGCG (Figure 7(d)), which indicated that the aromatic residues in hIAPP were getting unmovable or rigid, but the lag-time *t*_0_ of 74 ± 1 min corresponding to the nucleation rate with EGCG was smaller than that of 69 ± 1 min without EGCG; meanwhile the elongation rate *k* corresponding to the formation rate of rigid state was reduced from 0.107 ± 0.007 min^−1^ without EGCG to 0.051 ± 0.003 min^−1^ with EGCG on (1) (Table S8). These results indicated that EGCG could also inhibit the formation of aggregates of hIAPP.

**Figure 8:**
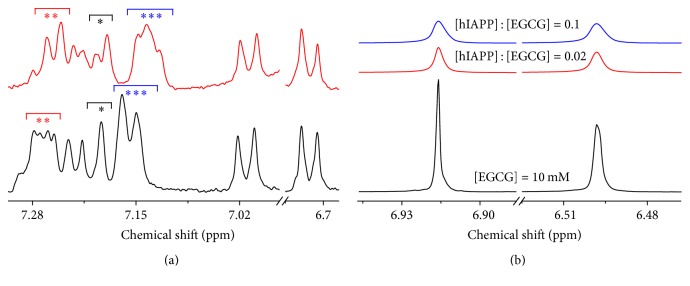
Interaction between hIAPP and EGCG. (a) Aromatic regions of ^1^H NMR spectra of 100 *μ*M hIAPP with (top) and without (bottom) 1 mM EGCG. One (*∗*), two (*∗∗*), and three (*∗∗∗*) asterisks represent *H*_*δ*_ of His18, *H*_*ε*_, and *H*_*ζ*_ of Phe15/Phe23 residues, respectively. (b) ^1^H NMR spectra of 1 mM EGCG in ratio of [hIAPP] : [EGCG] of 0 (bottom), 0.02 (medial), and 0.1 (top) *μ*M hIAPP within chemical shift from 6.47 to 6.95 ppm in D_2_O under 25°C.

Moreover, in order to reveal the interaction between hIAPP and EGCG in more detail on molecular level, ^1^H NMR was measured when 100 *μ*M hIAPP was incubated with or without high concentration of EGCG at 1 mM as shown in Figure 8(a). It is found that addition of EGCG significantly changed the peak features from 6.68 to 7.31 ppm which were assigned to the resonances of aromatic residues of hIAPP. The singlet *H*_*δ*_ resonance (symbol of *∗*) of His18 residue from 7.17 to 7.21 ppm [51] was split into doublet; the quartet *H*_*ε*_ resonances (symbol of *∗∗*) of phenylalanine 15 and phenylalanine 23 (Phe15/Phe23) residues from 7.24 to 7.28 ppm [55] were changed to doublet; and the doublet *H*_*ζ*_ resonances (symbol of *∗∗∗*) of Phe15/Phe23 residues from 7.11 to 7.17 ppm [55] were changed to triplet with up-field shift, while the chemical shifts and line widths of the resonances of *H*_*δ*_ from 7.21 to 7.24 ppm of Phe15/Phe23 residues [55] as well as *H*_*δ*_ from 6.71 to 6.73 ppm and *H*_*ε*_ from 7.00 to 7.02 ppm of tyrosine 37 (Tyr37) residue [51] were nearly unchanged. Meanwhile, the resonances of EGCG at *δ* = 6.92 and 6.50 ppm became broadened with the increased peak linewidth at half maximum height (*W*_1/2_) from 0.60 and 1.28 Hz to 2.32 and 2.60 Hz, respectively, as ratio of hIAPP to EGCG increased from 0 to 0.02 to 0.1 as shown in Figure 8(b) and Table S9. The results indicated the presence of direct *π*-*π* interaction between residues of Phe15/Phe23 as well as His18 of hIAPP and EGCG, leading to the linewidth broadened. Experimental and theoretical studies suggest that Phe23 can form *π*-*π* interactions with Phe23 residues from adjacent hIAPP monomers [56], which lead to the formation of amyloid. According to our previous work and literatures, the *π*-*π* stacking between EGCG and hIAPP could block competitively that between two hIAPP monomers [57], thus inhibiting the formation of hIAPP fibrils.

### 3.5. Influence of Al(III)/EGCG Complex on the Fibrillation and Aggregation of hIAPP

**Figure 9:**
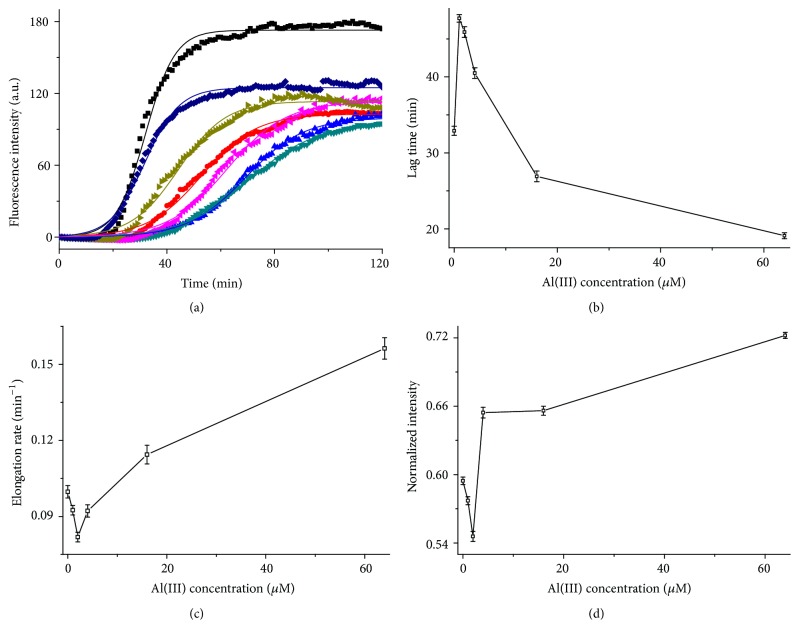
The influence of Al(III) on the EGCG-interfered hIAPP fibrillation. (a) Kinetics of 16 *μ*M hIAPP fibrillation without (■) or with 2 *μ*M EGCG interfered by addition of 0 (●), 1 (▲), 2 (▼), 4 (◂), 16 (▸), and 64 (◆) *μ*M Al(III) monitored by ThT fluorescence. The values represent mean (*n* = 3). Solid lines represent the curves fitted using (1). (b) Lag time, (c) elongation rate, and (d) the normalized maximum ThT fluorescence of hIAPP aggregation with 2 *μ*M EGCG on the Al(III) concentration. The intensity values were normalized to hIAPP alone data.

Furthermore, in terms of the results above about the effect of EGCG on the hIAPP fibrillation and aggregation, the influence of Al(III) on the EGCG-interfered process was investigated using 16 *μ*M hIAPP incubated with 2 *μ*M EGCG and then influenced by addition of Al(III) from 0 to 64 *μ*M, monitored by ThT fluorescence (Figure 9(a)). The detailed analysis for Figure 9(a) found that the fibrillation kinetics showed similar V-shaped trends on the Al(III) concentration at states of nucleation, elongation, and stabilization as shown in Figures 9(b)–9(d), respectively. It is found in Table S10 on (1) that the lag-time *t*_0_, that is, nucleation time, was increased when the concentration of Al(III) was increased from 0 to 2 *μ*M; that is, when the molar ratio of Al to EGCG was 1 : 1, the nucleation time was the longest, while it started reducing when the concentration of Al(III) was higher than 2 *μ*M (Figure 9(b)), which indicated that the concentration of Al(III) equivalent to that of EGCG could most efficiently delay the nucleation of hIAPP fibril. However, the opposite trends were found in the states of elongation (Figure 9(c)) and stabilization (Figure 9(d)) as the concentration of Al(III) was changed, which means the elongation rate *k* was the slowest and the maximum fibril amount *I*_max_ was the smallest when the molar ratio of Al to EGCG reached 1 : 1, and both of them were increased when the concentration of Al(III) was lower or higher than 2 *μ*M.

**Figure 10:**
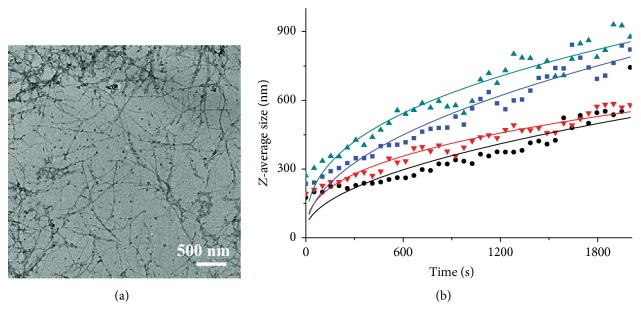
The influence of Al(III)/EGCG complex on the fibrillation and nucleation of hIAPP. (a) TEM image of 16 *μ*M hIAPP incubated with 2 *μ*M Al(III) and 2 *μ*M EGCG for 2 h. (b) Dependence of *Z*-average size on the nucleation time of 16 *μ*M hIAPP incubated without (■), and with 2 *μ*M EGCG (●), 2 *μ*M Al(III) (▲), and 2 *μ*M Al/EGCG in molar ration of 1 : 1 (▼) by DLS assay. Solid lines represent the curves fitted using (2).

The influence of Al(III)/EGCG complex in molar ratio of 1 : 1 on the fibrillation of hIAPP was investigated by TEM (Figure 10(a)). The fibrils in average diameter of 22 ± 6 nm and amorphous aggregates in average diameter of 37 ± 9 nm were observed in the hIAPP sample treated by Al(III)/EGCG complex, while only fibrils were found in hIAPP alone sample, which indicated that the Al(III)/EGCG complex could inhibit significantly the hIAPP fibrillation.

Moreover, the nucleation of 16 *μ*M hIAPP incubated with or without 2 *μ*M EGCG, 2 *μ*M Al(III), and 2 *μ*M Al(III)/EGCG complex in 0.7 mM PBS at pH 7.4 under 37°C was monitored by DLS technique (Figure 10(b)). The parameters of *D*_0_, *k*, and *n* were extracted from the fitted DLS curves on (2) and listed in Table S11. It is found in Table S11 that the hIAPP samples treated by EGCG and Al(III)/EGCG complex have similar *n* value of about 1.1, indicating that the nucleation models for these two EGCG-involved samples were similar. It is noted that both hIAPP alone and Al(III) treated hIAPP samples have another similar *n* value of about 1.5. The results indicated that the nucleation model of EGCG-interfered hIAPP was different from that of hIAPP and Al(III) treated one. The growth-rate-related parameter *k* of nucleus was in order as hIAPP treated by Al(III)/EGCG complex > by EGCG for *n* = 1.5 system and treated by Al(III) > by hIAPP alone for *n* = 1.1 system. Combining the morphologies of hIAPP observed by TEM, we suggest that the EGCG and Al(III)/EGCG complex could induce hIAPP forming the nontoxic amorphous nucleus or aggregates, while Al(III) could promote hIAPP forming the toxic fibrils.

It was reported that hIAPP has several positive charges at neutral pH values and is strongly attracted to a hydrophobic surface. hIAPP favours binding to surfaces via hydrophobic interaction more than electrostatic interaction in salt solution [58]. As EGCG molecule has many hydrophobic benzene rings, we supposed that hIAPP has good binding affinity to Al(III)/EGCG complex through *π*-*π* interaction, and the few positive charges of hIAPP cannot block this binding process in PBS solution. Al(III)/EGCG complex could bring more positive charges to hIAPP, thus leading to electrostatic repulsion between positive charged Phe23 residues in adjacent monomers stacked along the fibril axis, and then more effectively inhibited the self-assemble, fibrillation, and aggregation of hIAPP.

## 4. Conclusions

Our results suggested that Al(III) could stimulate the nucleation and fibrillation of hIAPP through His18-Al(III)-His18 cross-links between hIAPP monomers. EGCG could inhibit this process via the formation of hIAPP amorphous aggregates by *π*-*π* stacking between EGCG and aromatic residues of hIAPP. Al(III) could coordinate with O_8_ and C_9_ of EGCG to form Al(III)/EGCG complex in molar ratio of 1 : 1 as Al(EGCG)(H_2_O)_2_. Al(III)/EGCG complex could combine and positively charge Phe23 residues, thus could block the hIAPP fibrillation more efficiently than EGCG alone via electrostatic repulsion effect between adjacent hIAPP monomers, and even change the toxic fibrils into the nontoxic amorphous aggregates. Our findings may provide invaluable reference for the future development of novel inhibitors of toxic hIAPP fibrils in the management of diabetes.

## Supplementary Material

The major form of EGCG were monomer, dimer and trimer in EGCG aqueous solution, and the major molar ratios of EGCG to Al(III) were 1, 2, and 3 in Al(III)/EGCG equimolar mixture solution.

## References

[1] Taylor J. P., Hardy J., Fischbeck K. H. (2002). Toxic proteins in neurodegenerative disease. *Science*.

[2] Sacchettini J. C., Kelly J. W. (2002). Therapeutic strategies for human amyloid diseases. *Nature Reviews Drug Discovery*.

[3] Mishra R., Bulic B., Sellin D., Jha S., Waldmann H., Winter R. (2008). Small-molecule inhibitors of islet amyloid polypeptide fibril formation. *Angewandte Chemie—International Edition*.

[4] Westermark P., Wernstedt C., Wilander E., Hayden D. W., O'Brien T. D., Johnson K. H. (1987). Amyloid fibrils in human insulinoma and islets of Langerhans of the diabetic cat are derived from a neuropeptide-like protein also present in normal islet cells. *Proceedings of the National Academy of Sciences of the United States of America*.

[5] Brender J. R., Salamekh S., Ramamoorthy A. (2012). Membrane disruption and early events in the aggregation of the diabetes related peptide IAPP from a molecular perspective. *Accounts of Chemical Research*.

[6] Ritzel R. A., Meier J. J., Lin C.-Y., Veldhuis J. D., Butler P. C. (2007). Human islet amyloid polypeptide oligomers disrupt cell coupling, induce apoptosis, and impair insulin secretion in isolated human islets. *Diabetes*.

[7] Lin C.-Y., Gurlo T., Kayed R. (2007). Toxic human islet amyloid polypeptide (h-IAPP) oligomers are intracellular, and vaccination to induce anti-toxic oligomer antibodies does not prevent h-IAPP-induced *β*-cell apoptosis in h-IAPP transgenic mice. *Diabetes*.

[8] Westermark P., Andersson A., Westermark G. T. (2011). Islet amyloid polypeptide, islet amyloid, and diabetes mellitus. *Physiological Reviews*.

[9] Alexandrescu A. T. (2005). Amyloid accomplices and enforcers. *Protein Science*.

[10] Brender J. R., Hartman K., Nanga R. P. R. (2010). Role of zinc in human islet amyloid polypeptide aggregation. *Journal of the American Chemical Society*.

[11] Lee J. C., Gray H. B., Winkler J. R. (2008). Copper(II) binding to *α*-synuclein, the Parkinson's protein. *Journal of the American Chemical Society*.

[12] Casas S., Novials A., Reimann F., Gomis R., Gribble F. M. (2008). Calcium elevation in mouse pancreatic beta cells evoked by extracellular human islet amyloid polypeptide involves activation of the mechanosensitive ion channel TRPV4. *Diabetologia*.

[13] Savelieff M. G., Detoma A. S., Derrick J. S., Lim M. H. (2014). The ongoing search for small molecules to study metal-associated amyloid-*β* species in Alzheimer's disease. *Accounts of Chemical Research*.

[14] Zatta P., Lucchini R., Van Rensburg S. J., Taylor A. (2003). The role of metals in neurodegenerative processes: aluminum, manganese, and zinc. *Brain Research Bulletin*.

[15] Yao T., Jiang T., Pan D., Xu Z.-X., Zhou P. (2014). Effect of Al(III) and curcumin on silk fibroin conformation and aggregation morphology. *RSC Advances*.

[16] Flores C. R., Puga M. P., Wrobel K., Garay Sevilla M. E., Wrobel K. (2011). Trace elements status in diabetes mellitus type 2: possible role of the interaction between molybdenum and copper in the progress of typical complications. *Diabetes Research and Clinical Practice*.

[17] Exley C., House E., Patel T., Wu L., Fraser P. E. (2010). Human pro-islet amyloid polypeptide (ProIAPP1–48) forms amyloid fibrils and amyloid spherulites *in vitro*. *Journal of Inorganic Biochemistry*.

[18] Naiki H., Higuchi K., Hosokawa M., Takeda T. (1989). Fluorometric determination of amyloid fibrils in vitro using the fluorescent dye, thioflavine T. *Analytical Biochemistry*.

[19] Patel H. R., Pithadia A. S., Brender J. R., Fierke C. A., Ramamoorthy A. (2014). In search of aggregation pathways of IAPP and other amyloidogenic proteins: finding answers through NMR spectroscopy. *Journal of Physical Chemistry Letters*.

[20] Levy M., Porat Y., Bacharach E., Shalev D. E., Gazit E. (2008). Phenolsulfonphthalein, but not phenolphthalein, inhibits amyloid fibril formation: implications for the modulation of amyloid self-assembly. *Biochemistry*.

[21] Meng X. Y., Munishkina L. A., Fink A. L., Uversky V. N. (2010). Effects of various flavonoids on the *α*-synuclein fibrillation process. *Parkinson's Disease*.

[22] Mishra A., Misra A., Sri Vaishnavi T. (2013). Conformationally restricted short peptides inhibit human islet amyloid polypeptide (hIAPP) fibrillization. *Chemical Communications*.

[23] Porat Y., Mazor Y., Efrat S., Gazit E. (2004). Inhibition of islet amyloid polypeptide fibril formation: a potential role for heteroaromatic interactions. *Biochemistry*.

[24] Hu J., Yu Y.-P., Cui W. (2010). Cyclen-hybrid compound captures copper to protect INS-1 cells from islet amyloid polypeptide cytotoxicity by inhibiting and lysing effects. *Chemical Communications*.

[25] Jeong K., Cho H. R., Choi S. H., Park Y., Chae P. S. (2012). Protective effects of cleavage agents on INS-1 cells against h-IAPP-induced apoptosis. *Chemical Communications*.

[26] Jiang T., Zhi X.-L., Zhang Y.-H., Pan L.-F., Zhou P. (2012). Inhibitory effect of curcumin on the Al(III)-induced A*β*42 aggregation and neurotoxicity in vitro. *Biochimica et Biophysica Acta—Molecular Basis of Disease*.

[27] Meng F. L., Abedini A., Plesner A., Verchere C. B., Raleigh D. P. (2010). The flavanol (−)-epigallocatechin 3-gallate inhibits amyloid formation by islet amyloid polypeptide, disaggregates amyloid fibrils, and protects cultured cells against IAPP-induced toxicity. *Biochemistry*.

[28] Palhano F. L., Lee J., Grimster N. P., Kelly J. W. (2013). Toward the molecular mechanism(s) by which EGCG treatment remodels mature amyloid fibrils. *Journal of the American Chemical Society*.

[29] Cao P., Raleigh D. P. (2012). Analysis of the inhibition and remodeling of islet amyloid polypeptide amyloid fibers by flavanols. *Biochemistry*.

[30] Liu F.-F., Dong X.-Y., He L., Middelberg A. P. J., Sun Y. (2011). Molecular insight into conformational transition of amyloid *β*-peptide 42 inhibited by (−)-epigallocatechin-3-gallate probed by molecular simulations. *The Journal of Physical Chemistry B*.

[31] Popovych N., Brender J. R., Soong R. (2012). Site specific interaction of the polyphenol EGCG with the SEVI amyloid precursor peptide PAP(248-286). *Journal of Physical Chemistry B*.

[32] Liu Y., Liu Y., Wang S., Dong S., Chang P., Jiang Z. (2015). Structural characteristics of (-)-epigallocatechin-3-gallate inhibiting amyloid A*β*42 aggregation and remodeling amyloid fibers. *RSC Advances*.

[33] Bieschke J., Russ J., Friedrich R. P. (2010). EGCG remodels mature *α*-synuclein and amyloid-*β* fibrils and reduces cellular toxicity. *Proceedings of the National Academy of Sciences of the United States of America*.

[34] Ehrnhoefer D. E., Bieschke J., Boeddrich A. (2008). EGCG redirects amyloidogenic polypeptides into unstructured, off-pathway oligomers. *Nature Structural and Molecular Biology*.

[35] Suzuki Y., Brender J. R., Hartman K., Ramamoorthy A., Marsh E. N. G. (2012). Alternative pathways of human islet amyloid polypeptide aggregation distinguished by ^19^F nuclear magnetic resonance-detected kinetics of monomer consumption. *Biochemistry*.

[36] Hyung S. J., DeToma A. S., Brender J. R. (2013). Insights into antiamyloidogenic properties of the green tea extract (−)-epigallocatechin-3-gallate toward metal-associated amyloid-*β* species. *Proceedings of the National Academy of Sciences of the United States of America*.

[37] Mandel S. A., Avramovich-Tirosh Y., Reznichenko L. (2005). Multifunctional activities of green tea catechins in neuroprotection: modulation of cell survival genes, iron-dependent oxidative stress and PKC signaling pathway. *NeuroSignals*.

[38] Woehrle G. H., Hutchison J. E., Özkar S., Finke R. G. (2006). Analysis of nanoparticle transmission electron microscopy data using a public- domain image-processing program, Image. *Turkish Journal of Chemistry*.

[39] de León Y. P., Pichardo-Molina J. L., Ochoa N. A. (2014). Growth kinetics of concave nanocubes studied by optical coherence tomography. *Plasmonics*.

[40] Zhao Y., Truhlar D. G. (2006). A new local density functional for main-group thermochemistry, transition metal bonding, thermochemical kinetics, and noncovalent interactions. *Journal of Chemical Physics*.

[41] Zhao Y., Truhlar D. G. (2008). The M06 suite of density functionals for main group thermochemistry, thermochemical kinetics, noncovalent interactions, excited states, and transition elements: two new functionals and systematic testing of four M06-class functionals and 12 other functionals. *Theoretical Chemistry Accounts*.

[42] Scalmani G., Frisch M. J. (2010). Continuous surface charge polarizable continuum models of solvation. I. General formalism. *Journal of Chemical Physics*.

[43] Marenich A. V., Cramer C. J., Truhlar D. G. (2009). Universal solvation model based on solute electron density and on a continuum model of the solvent defined by the bulk dielectric constant and atomic surface tensions. *The Journal of Physical Chemistry B*.

[44] Inoue M. B., Inoue M., Fernando Q., Valcic S., Timmermann B. N. (2002). Potentiometric and ^1^H NMR studies of complexation of Al3+ with (–)-epigallocatechin gallate, a major active constituent of green tea. *Journal of Inorganic Biochemistry*.

[45] Beretta G., Furlanetto S., Regazzoni L., Zarrella M., Facino R. M. (2008). Quenching of *α*,*β*-unsaturated aldehydes by green tea polyphenols: HPLC-ESI-MS/MS studies. *Journal of Pharmaceutical and Biomedical Analysis*.

[46] Mukherjee A., Morales-Scheihing D., Butler P. C., Soto C. (2015). Type 2 diabetes as a protein misfolding disease. *Trends in Molecular Medicine*.

[47] Jarrett J. T., Lansbury P. T. (1993). Seeding “one-dimensional crystallization” of amyloid: a pathogenic mechanism in Alzheimer's disease and scrapie?. *Cell*.

[48] Linse B., Linse S. (2011). Monte Carlo simulations of protein amyloid formation reveal origin of sigmoidal aggregation kinetics. *Molecular BioSystems*.

[49] Mishra R., Geyer M., Winter R. (2009). NMR spectroscopic investigation of early events in IAPP amyloid fibril formation. *ChemBioChem*.

[50] Engel M. F. M., Khemtémourian L., Kleijer C. C. (2008). Membrane damage by human islet amyloid polypeptide through fibril growth at the membrane. *Proceedings of the National Academy of Sciences of the United States of America*.

[51] Valiente-Gabioud A. A., Torres-Monserrat V., Molina-Rubino L., Binolfi A., Griesinger C., Fernández C. O. (2012). Structural basis behind the interaction of Zn^2+^ with the protein *α*-synuclein and the A*β* peptide: a comparative analysis. *Journal of Inorganic Biochemistry*.

[52] Brender J. R., Lee E. L., Cavitt M. A., Gafni A., Steel D. G., Ramamoorthy A. (2008). Amyloid fiber formation and membrane disruption are separate processes localized in two distinct regions of IAPP, the type-2-diabetes-related peptide. *Journal of the American Chemical Society*.

[53] Brender J. R., Hartman K., Reid K. R., Kennedy R. T., Ramamoorthy A. (2008). A single mutation in the nonamyloidogenic region of islet amyloid polypeptide greatly reduces toxicity. *Biochemistry*.

[54] Salamekh S., Brender J. R., Hyung S.-J. (2011). A two-site mechanism for the inhibition of IAPP amyloidogenesis by zinc. *Journal of Molecular Biology*.

[55] Takeda M., Ono A. M., Terauchi T., Kainosho M. (2010). Application of SAIL phenylalanine and tyrosine with alternative isotope-labeling patterns for protein structure determination. *Journal of Biomolecular NMR*.

[56] Gazit E. (2002). A possible role for pi-stacking in the self-assembly of amyloid fibrils. *The FASEB Journal*.

[57] Huang R., Vivekanandan S., Brender J. R., Abe Y., Naito A., Ramamoorthy A. (2012). NMR characterization of monomeric and oligomeric conformations of human calcitonin and its interaction with EGCG. *Journal of Molecular Biology*.

[58] Jeworrek C., Hollmann O., Steitz R., Winter R., Czeslik C. (2009). Interaction of IAPP and insulin with model interfaces studied using neutron reflectometry. *Biophysical Journal*.

