# Supplementary material for: Influence of Aluminium and EGCG on Fibrillation and Aggregation of Human Islet Amyloid Polypeptide
Source: J Diabetes Res. 2016 Dec 15;2016:1867059. doi: 10.1155/2016/1867059 (PMC5198260; doi:10.1155/2016/1867059)
Supplement: Supplementary file 1 — The major form of EGCG were monomer, dimer and trimer in EGCG aqueous solution, and the major molar ratios of EGCG to Al(III) were 1, 2, and 3 in Al(III)/EGCG equimolar mixture solution. [file 1867059.f1.pdf]

Graphical abstracts for Table of Contents

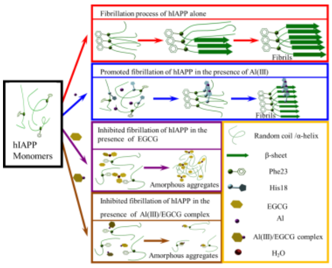

hIAPP monomers self-assemble into fibrils. EGCG efficiently inhibits this process, and even can inhibit Al(III)-induced fibrillation via Al(III)/EGCG complex of Al(EGCG)$(H_2O)_2$.

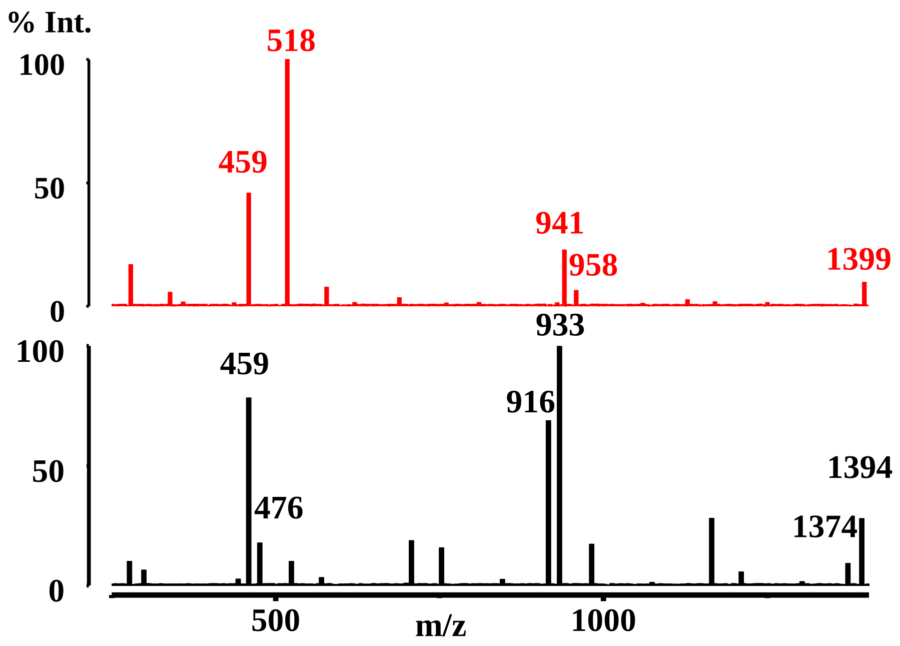

Fig. S1 ESI mass spectra of 10 mM EGCG without (bottom) and with (top) 10 mM Al(III).

Table S1 Assignments of ESI mass spectra of EGCG and Al(III)/EGCG complex[a]

EGCG		Al(III)/EGCG	
m/z	ion	m/z	ion
459	H^+E	459	H^+E
476	H^+EW	518	$Al^{3+}(H^+)_{-2}EW_2$
916	H^+E_2	941	$Al^{3+}(H^+)_{-2}E$
933	H^+E_2W	958	$Al^{3+}(H^+)_{-2}E_2W$
1374	H^+E_3	1399	$Al^{3+}(H^+)_{-2}E_3$
1394	Na^+E_3		

[a]E and W represent EGCG and water, respectively.

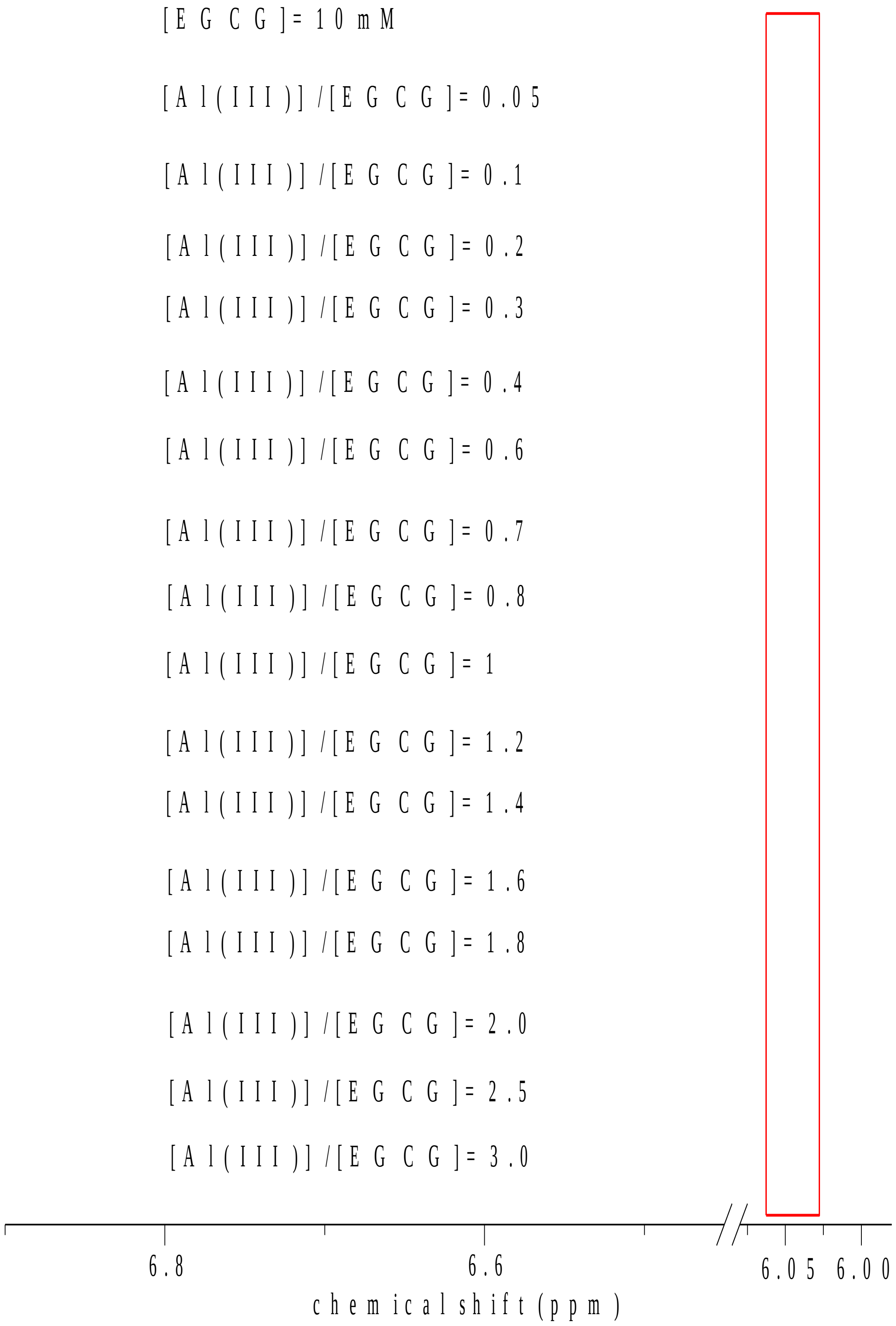

Fig. S2 ^{1}H NMR spectra of 10 mM EGCG in the presence of Al(III) at various concentrations from 0 to 30 mM. Intensity of peak at 6.04 ppm decreased with the titration of Al(III)

Table S2 Assignments of ^{1}H NMR spectra of EGCG and Al(III)/EGCG complex

EGCG		Al(III)/EGCG	
δ (ppm)	sites	δ (ppm)	sites
6.92	H-3’’ and H-7’’	6.92	H-3’’ and H-7’’
6.50	H-2’ and H-6’	6.80	H-3’’ and H-7’’
6.04	H-9	6.75	H-3’’ and H-7’’
6.02	H-7	6.55	H-2’ and H-6’
2.77-2.95	H-4	6.50	H-2’ and H-6’
		6.04	H-9
		6.02	H-7
		2.77-2.95	H-4

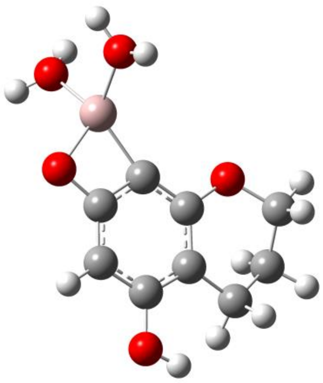

Charge = 1 Multiplicity = 1
E = -968.4423424 a.u.
C,-0.1181060603,2.1513427975,-0.0761798716
C,1.1964183484,1.7077932861,0.096382481
C,1.5739730081,0.3543043861,0.0429043402
C,0.5682708294,-0.5848548203,-0.2474120686
C,-0.7583133057,-0.2026944057,-0.4224441353
C,-1.0653646855,1.1686730201,-0.3214829309
H,3.6535359534,0.2034691085,-0.5089617051
C,2.9980612017,-0.0702527748,0.3283608391
C,2.2279917488,-2.2828294358,-0.4832676421
C,3.0660032715,-1.578054723,0.5621777369
O,0.843520921,-1.923981861,-0.3530032769
O,-2.4249333279,1.4117788893,-0.5104260233
O,2.1286540332,2.6830762872,0.3382399529
H,2.2531926805,-3.3676042206,-0.3742924697
H,3.3756697241,0.4535457055,1.2150146398
H,2.6774186639,-1.8288484641,1.5555297944
H,2.5624825656,-2.016483989,-1.4944304268
H,4.0982594212,-1.9327814485,0.5045519248
Al,-2.627016425,-0.3554541736,-0.7392537585
O,-3.9441865127,-1.0288009635,0.3403076343
H,-4.1068416981,-0.7096886745,1.2456787169
H,-4.3187951448,-1.9231722715,0.2464935676
H,3.021088199,2.3102327446,0.3799425566
H,-0.363286278,3.2061124546,-0.0202690067
O,-3.3891032752,-0.9734982513,-2.2820707995
H,-4.341877662,-1.1434046105,-2.3927253665
H,-2.9578618248,-0.980767582,-3.154668793

Fig. S3 Geometries and Cartesian coordinates of AE-1. Red, gray, pink and white balls represent oxygen, carbon, aluminum and hydrogen atoms respectively.

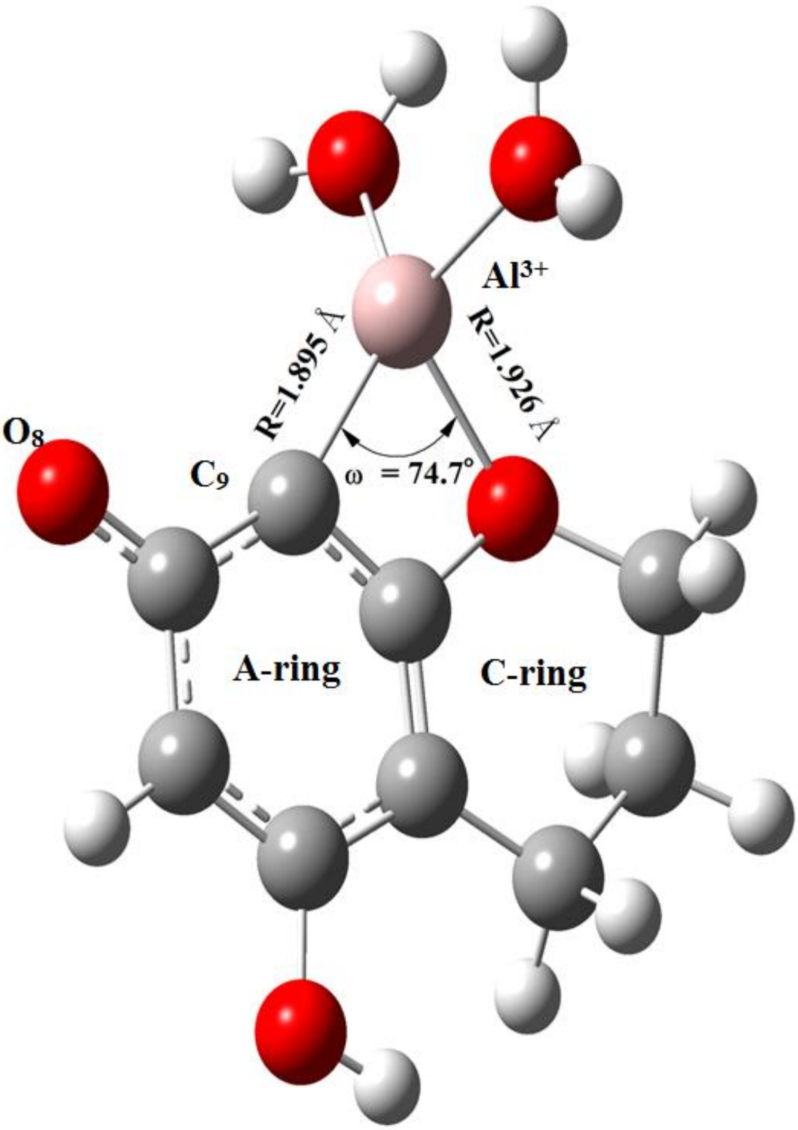

Fig. S4 Illustration of Al(EGCG)(H_2O)$_2$ complex 2 (AE-2). Red, gray, pink and white balls represent oxygen, carbon, aluminum and hydrogen atoms respectively. C_9 in A-ring and O atom in C-ring of EGCG form coordination bonds with aluminum.

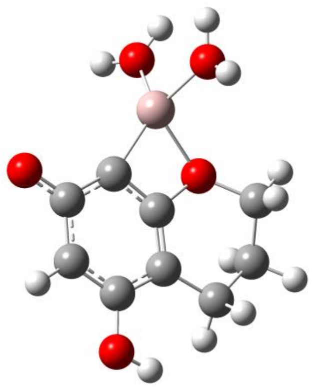

Charge = 1 Multiplicity = 1
E = -968.3992056 a.u.
C,-0.1387796648,2.1889338773,-0.0915244703
C,1.1774264082,1.7631186043,0.0218812299
C,1.5499330747,0.3976712855,0.0508439402
C,0.4563696685,-0.4266533516,-0.0626062979
C,-0.8925276905,-0.1066651385,-0.2113388868
C,-1.2397889819,1.2795877355,-0.2195223471
H,3.4810379674,0.0527749036,-0.8226810872
C,2.9584256309,-0.1417861217,0.1226397912
C,1.9086432475,-2.3689674212,-0.4422924512
C,2.9440132017,-1.6510219872,0.4010121601
O,0.595094082,-1.8531038364,-0.0617569802
O,-2.4536615418,1.6988182968,-0.3455598254
O,2.1384921672,2.7401748552,0.1015333835
H,1.8643124215,-3.4382171675,-0.2430427832
H,3.5287851114,0.3605832391,0.9122827913
H,2.7208466793,-1.8408622068,1.4560186488
H,2.0335051364,-2.1814336538,-1.5126886141
H,3.9226447903,-2.0861985379,0.1835509507
Al,-1.2629243899,-1.9427744117,-0.4187007024
O,-2.1628969011,-3.0457707769,0.7038461705
H,-2.556627032,-2.7641134705,1.5496550443
H,-2.3029113918,-4.0029289755,0.5830211464
H,3.0215348912,2.3503888179,0.1791242189
H,-0.3382069706,3.2567102977,-0.1026325722
O,-1.4646073375,-2.9486355657,-1.9187252427
H,-2.1267345359,-3.654806384,-2.0340297329
H,-1.0776575604,-2.724613416,-2.7847629821

Fig. S5 Geometries and Cartesian coordinates of AE-2. Red, gray, pink and white balls represent oxygen, carbon, aluminum and hydrogen atoms respectively.

Table S3 Binding free energies and their components from quantum chemical calculations for Al(III)/EGCG complexes (a.u.)

Contributions	AE-1	AE-2
$G_{(complex)}$	-968.27	-968.23
$G_{(H^+)}$	-0.23	-0.23
$G_{(Al^{3+})}$	-241.40	-241.40
$G_{(EGCG\bullet H_{-1})}$	-573.86	-573.86
$G_{(H_2O)}$	-76.41	-76.41
$\Delta G_{binding}$	-0.43	-0.39

Table S4 Fibrillation kinetic parameters of hIAPP on Al(III) concentration by ThT fluorescence assay

[Al] (μM)	I_{max}(A.U.)	$t_{1/2}$(min)	k(min^{-1})	t_0(min)	R
0	211.5 ±1.3	20.8 ±0.2	0.278 ±0.016	13.6 ±0.5	0.985
2	186.1 ±1.1	17.2 ±0.2	0.274 ±0.015	9.9 ±0.5	0.984
8	180.5 ±0.6	16.4 ±0.1	0.239 ±0.006	8.0 ±0.3	0.997
64	197.4 ±0.9	14.0 ±0.2	0.199 ±0.007	4.0 ±0.4	0.987

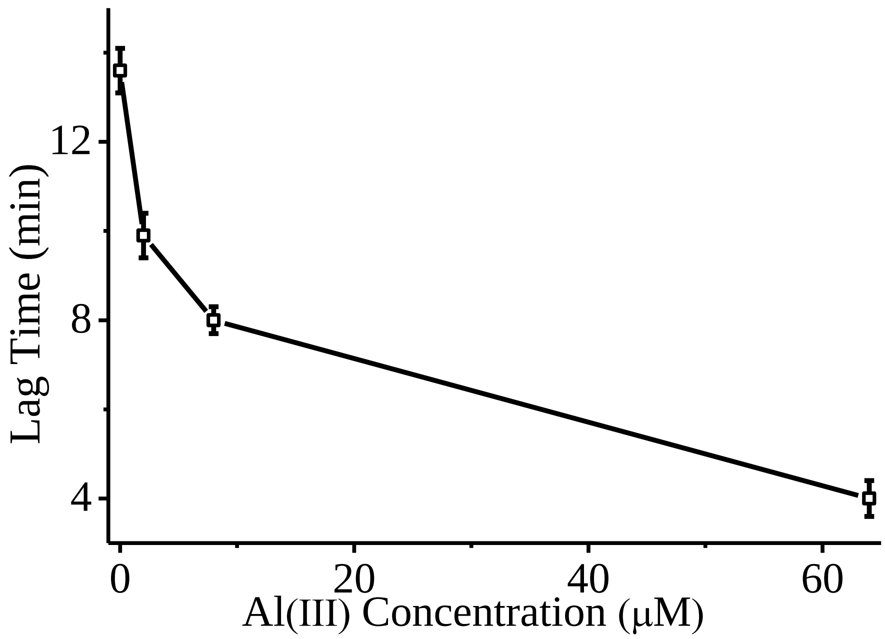

Fig. S6 Lag time (t_0) of 16 μM hIAPP fibrillation on Al(III) concentration

Table S5 Aggregation kinetic parameters of hIAPP with and without Al(III) by ^{1}H NMR

[Al] (μM)	I_{max}	$t_{1/2}$(min)	k(min^{-1})	t_0(min)	R
0	0.98±0.01	87.3±0.7	0.106±0.007	69±1	0.992
12.5	0.93±0.02	84.0±1.1	0.114±0.012	66±2	0.983

Table S6 Assignments of ^{1}H NMR spectra of aromatic resonances of hIAPP

δ (ppm)	residues	sites
7.24-7.28	Phe15/23	Hε
7.21-7.24	Phe15/23	Hδ
7.17-7.21	His18	Hδ
7.11-7.17	Phe15/23	Hζ
7.00-7.02	Tyr37	Hε
6.71-6.73	Tyr37	Hδ

Table S7 Fibrillation kinetic parameters of hIAPP on EGCG concentration by ThT fluorescence assay

[EGCG] (μM)	I_{max}(A.U.)	$t_{1/2}$(min)	k(min^{-1})	t_0(min)	R
0	186.9±3.1	23.0±0.9	0.122±0.004	6.6±1.8	0.983
2	136.0±3.0	39.6±1.1	0.069±0.004	10.5±2.0	0.995
8	101.0±0.9	38.4±0.4	0.102±0.003	18.7±0.8	0.995
16	69.8±0.5	35.3±0.4	0.113±0.004	17.6±0.7	0.986
32	42.2±0.4	23.4±0.4	0.169±0.010	11.6±0.8	0.986

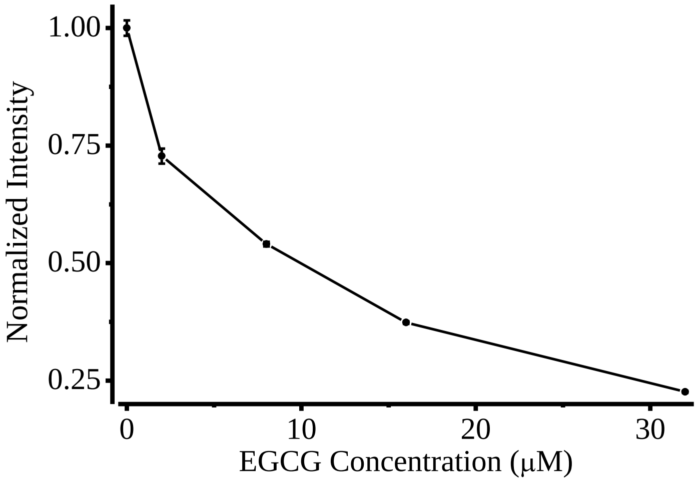

Fig. S7 The maximum ThT fluorescence intensity (I_{max}) of 16 μM hIAPP on EGCG concentration. The intensity values were normalized to the hIAPP alone data.

Table S8 Aggregation kinetic parameters of hIAPP with and without EGCG by ^{1}H NMR

[EGCG] (μM)	I_{max}	$t_{1/2}$(min)	k(min^{-1})	t_0(min)
0	0.98±0.01	87.3±0.7	0.106±0.007	69±1
12.5	1.06±0.01	113.2±1.1	0.051±0.003	74±2

Table S9 ^{1}H NMR peak line width at half maximum height ($W_{1/2}$) of EGCG on molar ratio of [hIAPP]/[EGCG]

[hIAPP]/[EGCG]	$W_{1/2}$ (Hz)	
	6.92 ppm	6.50 ppm
0	0.6	1.28
0.02	1.48	1.88
0.1	2.26	2.6

Table S10 Fibrillation kinetic parameters of 16 μM hIAPP with 2 μM EGCG on Al(III) concentration by ThT fluorescence assay

[Al] (μM)	I_{max}(A.U.)	$t_{1/2}$(min)	k(min^{-1})	t_0(min)
0	106.1±0.6	52.9±0.3	0.100±0.002	32.9±0.6
1	102.9±0.6	69.3±0.3	0.092±0.002	47.7±0.5
2	97.6±0.8	70.3±0.4	0.082±0.002	45.9±0.7
4	116.6±0.8	62.3±0.4	0.092±0.002	40.5±0.7
16	117.5±0.7	44.4±0.3	0.114±0.004	26.9±0.7
64	129.4±0.5	31.9±0.2	0.156±0.004	19.1±0.4

Table S11 Nucleation kinetic parameters of hIAPP by DLS assay

hIAPP co-incubated compounds	D_0(nm)	k	n
-	34.9±8.5	9.4±0.7	1.56±0.15
2 μM EGCG	12.8±4.4	31.9±1.4	1.11±0.06
2 μM Al(III)	18.8±7.2	19.5±0.8	1.44±0.08
2 μM Al(III)/EGCG (1:1)	20.2±7.5	43.8±2.0	1.17±0.05
